# 3-({[Bis(2-methyl­prop­yl)carbamo­thio­yl]amino}­carbon­yl)benzamide

**DOI:** 10.1107/S1600536813017455

**Published:** 2013-06-29

**Authors:** N. Selvakumaran, R. Karvembu, Seik Weng Ng, Edward R. T. Tiekink

**Affiliations:** a Department of Chemistry, National Institute of Technology, Tiruchirappalli 620 015, India; b Department of Chemistry, University of Malaya, 50603 Kuala Lumpur, Malaysia; c Chemistry Department, Faculty of Science, King Abdulaziz University, PO Box 80203 Jeddah, Saudi Arabia

## Abstract

In the title compound, C_17_H_25_N_3_O_2_S, the terminal and central amide groups are, respectively, twisted and coplanar with the attached benzene ring [O—C—C—C torsion angles = 22.7 (3) and 5.4 (3)°]. In the central part of the mol­ecule, the amide and thio­amide residues are approximately perpendicular [C—N—C—S torsion angle = −104.98 (18)°]. Supra­molecular layers with a zigzag topology are formed in the crystal packing by N—H⋯O, N—H⋯S and C—H⋯O inter­actions; these stack along *c*, being separated by hydro­phobic inter­actions.

## Related literature

For the preparation of bipodal acyl­thio­urea derivatives, see: Bourne *et al.* (2005 ▶). For a related structure, see: Selvakumaran *et al.* (2013 ▶).
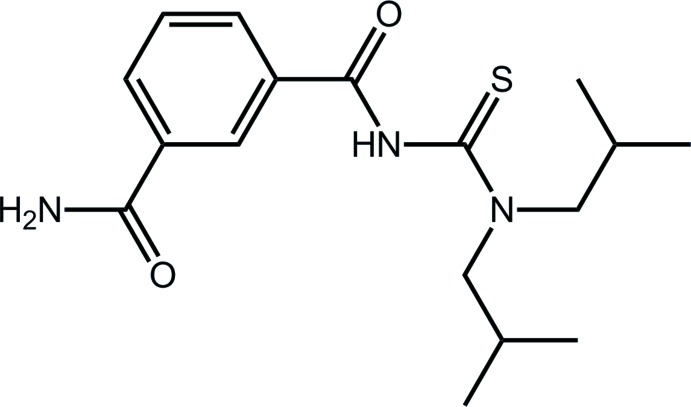


## Experimental

### 

#### Crystal data

C_17_H_25_N_3_O_2_S
*M* *_r_* = 335.46
Orthorhombic, 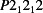

*a* = 13.9870 (4) Å
*b* = 15.7103 (4) Å
*c* = 8.5532 (3) Å
*V* = 1879.48 (10) Å^3^
*Z* = 4
Mo *K*α radiation
μ = 0.18 mm^−1^
*T* = 100 K
0.40 × 0.30 × 0.20 mm

#### Data collection

Agilent SuperNova Dual diffractometer with an Atlas detector
Absorption correction: multi-scan (*CrysAlis PRO*; Agilent, 2013 ▶) *T*_min_ = 0.930, *T*_max_ = 0.964
6635 measured reflections
4007 independent reflections
3694 reflections with *I* > 2σ(*I*)
*R*_int_ = 0.020

#### Refinement

*R*[*F* ^2^ > 2σ(*F* ^2^)] = 0.039
*wR*(*F* ^2^) = 0.097
*S* = 1.00
4007 reflections
218 parameters
30 restraints
H-atom parameters constrained
Δρ_max_ = 0.27 e Å^−3^
Δρ_min_ = −0.24 e Å^−3^
Absolute structure: Flack (1983) ▶, 1590 Friedel pairs
Flack parameter: −0.03 (8)

### 

Data collection: *CrysAlis PRO* (Agilent, 2013 ▶); cell refinement: *CrysAlis PRO*; data reduction: *CrysAlis PRO*; program(s) used to solve structure: *SHELXS97* (Sheldrick, 2008 ▶); program(s) used to refine structure: *SHELXL97* (Sheldrick, 2008 ▶); molecular graphics: *ORTEP-3 for Windows* (Farrugia, 2012 ▶) and *DIAMOND* (Brandenburg, 2006 ▶); software used to prepare material for publication: *publCIF* (Westrip, 2010 ▶).

## Supplementary Material

Crystal structure: contains datablock(s) global, I. DOI: 10.1107/S1600536813017455/hg5326sup1.cif

Structure factors: contains datablock(s) I. DOI: 10.1107/S1600536813017455/hg5326Isup2.hkl

Supplementary material file. DOI: 10.1107/S1600536813017455/hg5326Isup3.cml

Additional supplementary materials:  crystallographic information; 3D view; checkCIF report

## Figures and Tables

**Table 1:** Hydrogen-bond geometry (Å, °)

*D*—H⋯*A*	*D*—H	H⋯*A*	*D*⋯*A*	*D*—H⋯*A*
N1—H12⋯O2^i^	0.88	2.09	2.887 (2)	150
N2—H2⋯O1^ii^	0.88	1.97	2.797 (2)	155
N1—H11⋯S1^ii^	0.88	2.54	3.3908 (18)	163
C7—H7⋯O1^ii^	0.95	2.32	3.210 (2)	155

Symmetry codes: (i) 
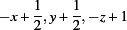
; (ii) 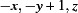.

**Fig. 1.**
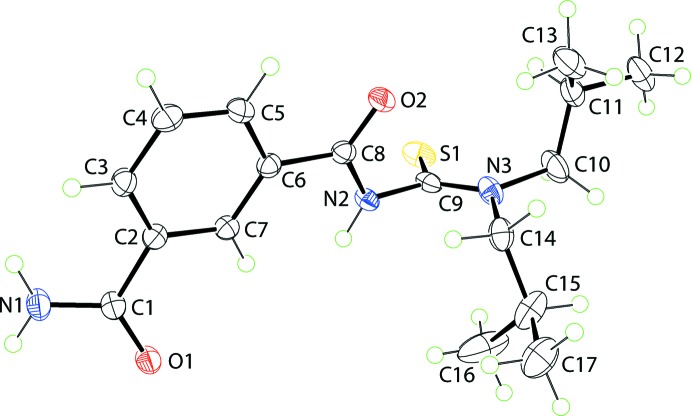
Molecular structure of (I) showing atom-labelling scheme and displacement ellipsoids at the 50% probability level.

**Fig. 2.**
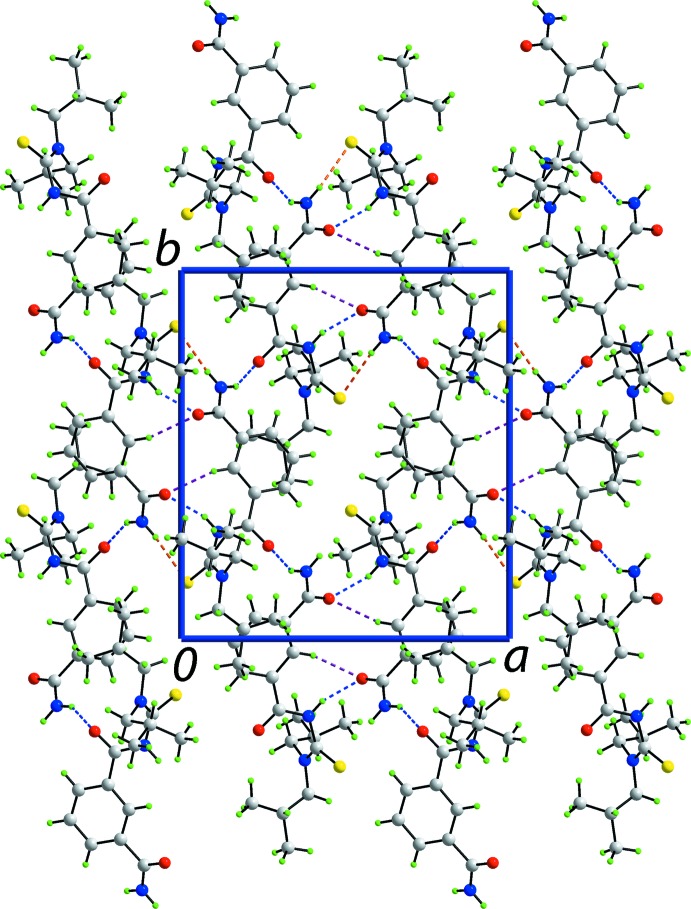
A plan view of the zigzag supramolecular layer in (I). The N—H···O, N—H···S and C—H···O interactions are shown as blue, orange and purple dashed lines, respectively.

**Fig. 3.**
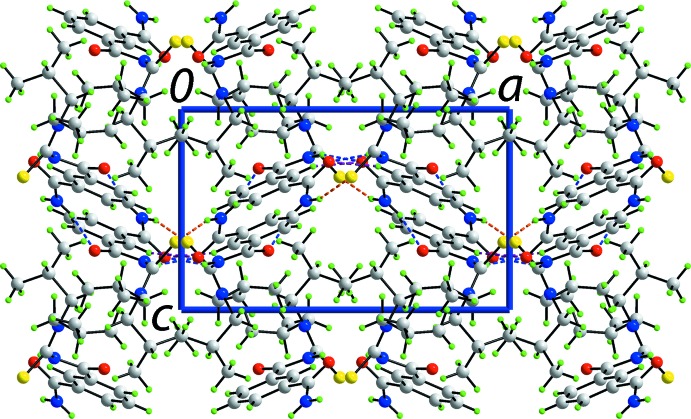
A view of the unit-cell contents in projection down the b axis in (I). The N—H···O, N—H···S and C—H···O interactions are shown as blue, orange and purple dashed lines, respectively.

## supplementary crystallographic information

### Comment

The title compound, (I), was obtained as a by-product in an attempt to prepare a bipodal acylthiourea derivative (Bourne *et al.*, 2005) from diisobutylamine, isophthaloyl dichloride and potassium thiocyanate in acetone. Crystals were grown from a solution of the compound in acetonitrile/dimethyl formamide mixture (1:1). In (I), Fig. 1, the terminal [O1—C1—C2—C7 torsion angle = 22.7 (3)°] and central [C5—C6—C8—O2 = 5.4 (3)°] amide substituents are twisted and co-planar with the attached benzene ring, respectively. A twist is also noted between the amide and adjacent thioamide residues as seen in the C8—N2—C9—S1 torsion angle of -104.98 (18)°. The methylpropyl substituents lie to either side and are approximately perpendicular to the C_3_N plane with the C9—N3—C10—C11/C11' (50:50 disorder in the isopropyl group) torsion angles being 126.0 (3) and 87.5 (3)°, respectively, and 100.4 (2)° for C9—N3—C14—C1. The aforementioned conformation matches that found in the accompanying paper (Selvakumaran *et al.*, 2013). In the crystal packing, supramolecular layers with a zigzag topology are formed by N—H···O, N—H···S and C—H···O interactions, Fig. 2 and Table 1. Layers stack along the *c* axis being separated by hydrophobic intertactions.

### Experimental

Isophthaloyl dichloride (2.0302 g, 10 mmol) dissolved in acetone (80 ml), was placed in a dropping funnel and added drop wise with stirring to potassium thiocyanate (1.9436 g, 20 mmol) dissolved in acetone (80 ml), under N_2_ atmosphere, in a three-necked round bottom flask. The mixture was heated to reflux for 30 minutes and then allowed to cool. A solution of diisobutylamine (2.2850 g, 20 mmol) in acetone (80 ml) was added drop wise from a dropping funnel to the reaction mixture and the resulting mixture was stirred for 2 h at room temperature. Then, hydrochloric acid (0.1 N, 300 ml) was added and the resulting white solid was filtered off, washed with water and dried *in vacuo*. Single crystals were grown at room temperature from acetonitrile/dimethyl formamide mixture (1:1) F T—IR (KBr): ν(NH_2_) 3217 & 3192, ν(N—H) 3405, ν(C═O) 1671 (with adjacent NH_2_), ν(C═O) 1652 (with adjacent NH), ν(C═C) 1593, ν(C═S) 1257 cm^-1^. UV-Visible (DMF): ν_max_; 264, 283, 363 nm.

### Refinement

Carbon-bound H-atoms were placed in calculated positions [C—H = 0.95 to 1.00 Å, *U*_iso_(H)= 1.2 to 1.5*U*_eq_(C)] and were included in the refinement in the riding model approximation. The amino H-atoms were similarly constrained [N—H = 0.88 Å, and with *U*_iso_(H)= 1.2*U*_eq_(N)]. One isopropyl arm is disordered; the disorder refined to exactly 0.5. The 1,2-related distances were restrained to 1.54±0.01 Å and the 1,3-related ones to 2.51±0.01 Å. The anisotropic displacement parameters of the primed atoms were set to those of the unprimed ones, and the anisotropic displacement parameters were restrained to be nearly isotropic.

### Crystal data

**Table:**

C_17_H_25_N_3_O_2_S	*F*(000) = 720
*M**_r_* = 335.46	*D*_x_ = 1.186 Mg m^−^^3^
Orthorhombic, *P*2_1_2_1_2	Mo *K*α radiation, λ = 0.71073 Å
Hall symbol: P 2 2ab	Cell parameters from 3818 reflections
*a* = 13.9870 (4) Å	θ = 2.4–29.3°
*b* = 15.7103 (4) Å	µ = 0.18 mm^−^^1^
*c* = 8.5532 (3) Å	*T* = 100 K
*V* = 1879.48 (10) Å^3^	Block, colourless
*Z* = 4	0.40 × 0.30 × 0.20 mm

### Data collection

**Table:**

Agilent SuperNova Dual diffractometer with an Atlas detector	4007 independent reflections
Radiation source: SuperNova (Mo) X-ray Source	3694 reflections with *I* > 2σ(*I*)
Mirror monochromator	*R*_int_ = 0.020
Detector resolution: 10.4041 pixels mm^-1^	θ_max_ = 27.5°, θ_min_ = 2.4°
ω scan	*h* = −17→18
Absorption correction: multi-scan (*CrysAlis PRO*; Agilent, 2013)	*k* = −17→20
*T*_min_ = 0.930, *T*_max_ = 0.964	*l* = −7→10
6635 measured reflections	

### Refinement

**Table:**

Refinement on *F*^2^	Secondary atom site location: difference Fourier map
Least-squares matrix: full	Hydrogen site location: inferred from neighbouring sites
*R*[*F*^2^ > 2σ(*F*^2^)] = 0.039	H-atom parameters constrained
*wR*(*F*^2^) = 0.097	*w* = 1/[σ^2^(*F*_o_^2^) + (0.0461*P*)^2^ + 0.7082*P*] where *P* = (*F*_o_^2^ + 2*F*_c_^2^)/3
*S* = 1.00	(Δ/σ)_max_ = 0.001
4007 reflections	Δρ_max_ = 0.27 e Å^−^^3^
218 parameters	Δρ_min_ = −0.24 e Å^−^^3^
30 restraints	Absolute structure: Flack (1983), 1590 Friedel pairs
Primary atom site location: structure-invariant direct methods	Flack parameter: −0.03 (8)

### Special details

**Table:**

Geometry. All e.s.d.'s (except the e.s.d. in the dihedral angle between two l.s. planes) are estimated using the full covariance matrix. The cell e.s.d.'s are taken into account individually in the estimation of e.s.d.'s in distances, angles and torsion angles; correlations between e.s.d.'s in cell parameters are only used when they are defined by crystal symmetry. An approximate (isotropic) treatment of cell e.s.d.'s is used for estimating e.s.d.'s involving l.s. planes.

### Fractional atomic coordinates and isotropic or equivalent isotropic displacement parameters (Å^2^)

**Table:**

	*x*	*y*	*z*	*U*_iso_*/*U*_eq_	Occ. (<1)
S1	0.01768 (4)	0.15262 (3)	0.66311 (7)	0.03126 (14)	
O1	0.05091 (10)	0.60837 (8)	0.73835 (19)	0.0262 (3)	
O2	0.26459 (10)	0.25192 (9)	0.70500 (17)	0.0268 (3)	
N1	0.12011 (13)	0.68211 (11)	0.5438 (2)	0.0311 (4)	
H11	0.0791	0.7243	0.5533	0.037*	
H12	0.1655	0.6846	0.4727	0.037*	
N2	0.11244 (11)	0.28966 (10)	0.7599 (2)	0.0199 (3)	
H2	0.0705	0.3307	0.7742	0.024*	
N3	0.12403 (12)	0.17031 (10)	0.9200 (2)	0.0253 (4)	
C1	0.11351 (14)	0.61468 (12)	0.6368 (2)	0.0222 (4)	
C2	0.18533 (14)	0.54440 (12)	0.6160 (2)	0.0207 (4)	
C3	0.27479 (14)	0.55770 (13)	0.5504 (3)	0.0247 (4)	
H3	0.2921	0.6127	0.5136	0.030*	
C4	0.33900 (16)	0.49082 (14)	0.5385 (3)	0.0299 (5)	
H4	0.4006	0.5001	0.4948	0.036*	
C5	0.31354 (15)	0.41050 (13)	0.5902 (3)	0.0260 (4)	
H5	0.3578	0.3649	0.5817	0.031*	
C6	0.22343 (13)	0.39591 (12)	0.6546 (2)	0.0204 (4)	
C7	0.15980 (13)	0.46342 (12)	0.6693 (2)	0.0196 (4)	
H7	0.0989	0.4545	0.7157	0.024*	
C8	0.20241 (14)	0.30731 (12)	0.7075 (2)	0.0202 (4)	
C9	0.08818 (13)	0.20345 (12)	0.7909 (2)	0.0221 (4)	
C10	0.11303 (17)	0.07868 (12)	0.9522 (3)	0.0371 (6)	
H10A	0.0508	0.0592	0.9102	0.045*	0.500 (4)
H10B	0.1122	0.0697	1.0668	0.045*	0.500 (4)
H10C	0.0696	0.0722	1.0429	0.045*	0.500 (4)
H10D	0.0812	0.0522	0.8611	0.045*	0.500 (4)
C11	0.1901 (3)	0.0265 (3)	0.8831 (6)	0.0295 (8)	0.500 (4)
H11A	0.1766	0.0275	0.7683	0.035*	0.500 (4)
C12	0.1752 (7)	−0.0672 (4)	0.9301 (7)	0.0374 (13)	0.500 (4)
H12A	0.1078	−0.0765	0.9566	0.056*	0.500 (4)
H12B	0.2152	−0.0805	1.0209	0.056*	0.500 (4)
H12C	0.1930	−0.1042	0.8426	0.056*	0.500 (4)
C13	0.2851 (7)	0.0534 (13)	0.895 (3)	0.0506 (12)	0.500 (4)
H13A	0.2886	0.1149	0.8759	0.076*	0.500 (4)
H13B	0.3241	0.0234	0.8175	0.076*	0.500 (4)
H13C	0.3092	0.0409	1.0002	0.076*	0.500 (4)
C11'	0.2013 (3)	0.0304 (3)	0.9852 (6)	0.0295 (8)	0.50
H11'	0.2189	0.0473	1.0942	0.035*	0.500 (4)
C12'	0.1753 (7)	−0.0640 (4)	0.9979 (7)	0.0374 (13)	0.50
H12D	0.1074	−0.0696	1.0237	0.056*	0.500 (4)
H12E	0.2139	−0.0906	1.0801	0.056*	0.500 (4)
H12F	0.1881	−0.0922	0.8978	0.056*	0.500 (4)
C13'	0.2927 (7)	0.0473 (13)	0.889 (3)	0.0506 (12)	0.50
H13D	0.2991	0.1085	0.8696	0.076*	0.500 (4)
H13E	0.2883	0.0172	0.7886	0.076*	0.500 (4)
H13F	0.3485	0.0268	0.9467	0.076*	0.500 (4)
C14	0.16514 (15)	0.22155 (13)	1.0474 (3)	0.0274 (5)	
H14A	0.1832	0.2782	1.0063	0.033*	
H14B	0.2240	0.1935	1.0861	0.033*	
C15	0.09549 (17)	0.23284 (18)	1.1831 (3)	0.0410 (6)	
H15	0.0827	0.1756	1.2298	0.049*	
C16	0.00072 (19)	0.2703 (2)	1.1290 (3)	0.0612 (9)	
H16A	−0.0273	0.2335	1.0486	0.092*	
H16B	0.0116	0.3272	1.0854	0.092*	
H16C	−0.0431	0.2744	1.2181	0.092*	
C17	0.1423 (2)	0.28857 (19)	1.3076 (3)	0.0475 (7)	
H17A	0.2021	0.2622	1.3421	0.071*	
H17B	0.0989	0.2944	1.3971	0.071*	
H17C	0.1557	0.3449	1.2638	0.071*	

### Atomic displacement parameters (Å^2^)

**Table:**

	*U*^11^	*U*^22^	*U*^33^	*U*^12^	*U*^13^	*U*^23^
S1	0.0270 (3)	0.0254 (2)	0.0414 (3)	−0.0024 (2)	−0.0020 (2)	−0.0130 (2)
O1	0.0196 (6)	0.0223 (7)	0.0367 (9)	0.0022 (6)	0.0062 (6)	0.0054 (6)
O2	0.0231 (7)	0.0245 (7)	0.0329 (9)	0.0073 (6)	0.0058 (6)	0.0032 (6)
N1	0.0249 (9)	0.0254 (8)	0.0431 (12)	0.0041 (7)	0.0082 (8)	0.0128 (8)
N2	0.0163 (7)	0.0166 (7)	0.0268 (9)	0.0017 (6)	0.0003 (7)	−0.0011 (7)
N3	0.0212 (8)	0.0179 (8)	0.0370 (10)	−0.0007 (6)	−0.0017 (7)	0.0028 (7)
C1	0.0179 (9)	0.0216 (9)	0.0272 (11)	−0.0012 (8)	−0.0013 (8)	0.0026 (8)
C2	0.0189 (9)	0.0230 (9)	0.0202 (10)	0.0002 (8)	−0.0005 (7)	0.0013 (8)
C3	0.0226 (9)	0.0235 (9)	0.0280 (11)	−0.0006 (8)	0.0041 (8)	0.0051 (9)
C4	0.0213 (10)	0.0330 (11)	0.0354 (12)	−0.0006 (9)	0.0101 (10)	0.0022 (10)
C5	0.0233 (10)	0.0241 (10)	0.0307 (12)	0.0052 (8)	0.0055 (9)	0.0014 (9)
C6	0.0200 (9)	0.0227 (9)	0.0186 (9)	0.0006 (7)	−0.0002 (8)	0.0008 (8)
C7	0.0176 (8)	0.0218 (9)	0.0196 (9)	−0.0002 (7)	−0.0002 (8)	0.0001 (8)
C8	0.0213 (9)	0.0221 (9)	0.0173 (10)	0.0021 (8)	−0.0012 (7)	−0.0019 (7)
C9	0.0174 (8)	0.0176 (9)	0.0312 (12)	0.0027 (7)	0.0045 (8)	−0.0034 (8)
C10	0.0390 (13)	0.0167 (10)	0.0556 (16)	0.0022 (9)	0.0046 (12)	0.0065 (10)
C11	0.0374 (17)	0.0237 (13)	0.0274 (17)	0.0075 (12)	−0.0093 (16)	0.0005 (16)
C12	0.0503 (17)	0.0218 (13)	0.040 (4)	0.0101 (12)	−0.019 (4)	−0.004 (3)
C13	0.0451 (18)	0.042 (2)	0.065 (2)	0.0223 (19)	0.0127 (18)	0.0127 (18)
C11'	0.0374 (17)	0.0237 (13)	0.0274 (17)	0.0075 (12)	−0.0093 (16)	0.0005 (16)
C12'	0.0503 (17)	0.0218 (13)	0.040 (4)	0.0101 (12)	−0.019 (4)	−0.004 (3)
C13'	0.0451 (18)	0.042 (2)	0.065 (2)	0.0223 (19)	0.0127 (18)	0.0127 (18)
C14	0.0238 (10)	0.0267 (10)	0.0318 (12)	−0.0025 (8)	−0.0064 (9)	0.0066 (9)
C15	0.0399 (13)	0.0582 (16)	0.0249 (12)	−0.0110 (12)	−0.0014 (11)	0.0082 (11)
C16	0.0314 (13)	0.117 (3)	0.0349 (15)	0.0081 (16)	0.0033 (11)	−0.0206 (16)
C17	0.0487 (15)	0.0643 (17)	0.0297 (14)	−0.0072 (13)	−0.0058 (12)	0.0027 (12)

### Geometric parameters (Å, º)

**Table:**

S1—C9	1.675 (2)	C11—C13	1.398 (13)
O1—C1	1.237 (2)	C11—C12	1.540 (7)
O2—C8	1.230 (2)	C11—H11A	1.0000
N1—C1	1.328 (3)	C12—H12A	0.9800
N1—H11	0.8800	C12—H12B	0.9800
N1—H12	0.8800	C12—H12C	0.9800
N2—C8	1.364 (2)	C13—H13A	0.9800
N2—C9	1.421 (2)	C13—H13B	0.9800
N2—H2	0.8800	C13—H13C	0.9800
N3—C9	1.320 (3)	C11'—C13'	1.545 (9)
N3—C14	1.472 (3)	C11'—C12'	1.531 (6)
N3—C10	1.474 (2)	C11'—H11'	1.0000
C1—C2	1.503 (3)	C12'—H12D	0.9800
C2—C3	1.387 (3)	C12'—H12E	0.9800
C2—C7	1.398 (3)	C12'—H12F	0.9800
C3—C4	1.386 (3)	C13'—H13D	0.9800
C3—H3	0.9500	C13'—H13E	0.9800
C4—C5	1.384 (3)	C13'—H13F	0.9800
C4—H4	0.9500	C14—C15	1.525 (3)
C5—C6	1.394 (3)	C14—H14A	0.9900
C5—H5	0.9500	C14—H14B	0.9900
C6—C7	1.390 (3)	C15—C17	1.526 (4)
C6—C8	1.493 (3)	C15—C16	1.523 (4)
C7—H7	0.9500	C15—H15	1.0000
C10—C11'	1.475 (4)	C16—H16A	0.9800
C10—C11	1.478 (5)	C16—H16B	0.9800
C10—H10A	0.9900	C16—H16C	0.9800
C10—H10B	0.9900	C17—H17A	0.9800
C10—H10C	0.9900	C17—H17B	0.9800
C10—H10D	0.9900	C17—H17C	0.9800
			
C1—N1—H11	120.0	C11—C12—H12A	109.5
C1—N1—H12	120.0	C11—C12—H12B	109.5
H11—N1—H12	120.0	H12A—C12—H12B	109.5
C8—N2—C9	118.37 (16)	C11—C12—H12C	109.5
C8—N2—H2	120.8	H12A—C12—H12C	109.5
C9—N2—H2	120.8	H12B—C12—H12C	109.5
C9—N3—C14	123.52 (16)	C11—C13—H13A	109.5
C9—N3—C10	120.15 (19)	C11—C13—H13B	109.5
C14—N3—C10	115.87 (19)	H13A—C13—H13B	109.5
O1—C1—N1	122.25 (18)	C11—C13—H13C	109.5
O1—C1—C2	119.81 (17)	H13A—C13—H13C	109.5
N1—C1—C2	117.94 (18)	H13B—C13—H13C	109.5
C3—C2—C7	119.97 (18)	C10—C11'—C13'	120.2 (8)
C3—C2—C1	122.69 (17)	C10—C11'—C12'	108.2 (4)
C7—C2—C1	117.33 (17)	C13'—C11'—C12'	113.6 (8)
C2—C3—C4	120.00 (19)	C10—C11'—H11'	104.4
C2—C3—H3	120.0	C13'—C11'—H11'	104.4
C4—C3—H3	120.0	C12'—C11'—H11'	104.4
C5—C4—C3	120.08 (19)	C11'—C12'—H12D	109.5
C5—C4—H4	120.0	C11'—C12'—H12E	109.5
C3—C4—H4	120.0	H12D—C12'—H12E	109.5
C4—C5—C6	120.58 (19)	C11'—C12'—H12F	109.5
C4—C5—H5	119.7	H12D—C12'—H12F	109.5
C6—C5—H5	119.7	H12E—C12'—H12F	109.5
C7—C6—C5	119.28 (18)	C11'—C13'—H13D	109.5
C7—C6—C8	123.91 (17)	C11'—C13'—H13E	109.5
C5—C6—C8	116.80 (17)	H13D—C13'—H13E	109.5
C6—C7—C2	120.08 (18)	C11'—C13'—H13F	109.5
C6—C7—H7	120.0	H13D—C13'—H13F	109.5
C2—C7—H7	120.0	H13E—C13'—H13F	109.5
O2—C8—N2	120.92 (18)	N3—C14—C15	112.17 (18)
O2—C8—C6	120.99 (17)	N3—C14—H14A	109.2
N2—C8—C6	118.08 (16)	C15—C14—H14A	109.2
N3—C9—N2	116.19 (17)	N3—C14—H14B	109.2
N3—C9—S1	125.57 (15)	C15—C14—H14B	109.2
N2—C9—S1	118.24 (16)	H14A—C14—H14B	107.9
C11'—C10—N3	116.8 (2)	C14—C15—C17	108.9 (2)
N3—C10—C11	113.0 (2)	C14—C15—C16	111.7 (2)
N3—C10—H10A	109.0	C17—C15—C16	111.3 (2)
C11—C10—H10A	109.0	C14—C15—H15	108.3
N3—C10—H10B	109.0	C17—C15—H15	108.3
C11—C10—H10B	109.0	C16—C15—H15	108.3
H10A—C10—H10B	107.8	C15—C16—H16A	109.5
C11'—C10—H10C	108.1	C15—C16—H16B	109.5
N3—C10—H10C	108.1	H16A—C16—H16B	109.5
C11'—C10—H10D	108.1	C15—C16—H16C	109.5
N3—C10—H10D	108.1	H16A—C16—H16C	109.5
H10C—C10—H10D	107.3	H16B—C16—H16C	109.5
C13—C11—C10	119.7 (9)	C15—C17—H17A	109.5
C13—C11—C12	113.5 (9)	C15—C17—H17B	109.5
C10—C11—C12	109.1 (4)	H17A—C17—H17B	109.5
C13—C11—H11A	104.2	C15—C17—H17C	109.5
C10—C11—H11A	104.2	H17A—C17—H17C	109.5
C12—C11—H11A	104.2	H17B—C17—H17C	109.5
			
O1—C1—C2—C3	−155.8 (2)	C10—N3—C9—N2	−172.35 (18)
N1—C1—C2—C3	24.2 (3)	C14—N3—C9—S1	−164.30 (16)
O1—C1—C2—C7	22.7 (3)	C10—N3—C9—S1	7.6 (3)
N1—C1—C2—C7	−157.3 (2)	C8—N2—C9—N3	75.0 (2)
C7—C2—C3—C4	−0.3 (3)	C8—N2—C9—S1	−104.98 (18)
C1—C2—C3—C4	178.1 (2)	C9—N3—C10—C11'	126.0 (3)
C2—C3—C4—C5	0.8 (4)	C14—N3—C10—C11'	−61.5 (3)
C3—C4—C5—C6	0.0 (4)	C9—N3—C10—C11	87.5 (3)
C4—C5—C6—C7	−1.2 (3)	C14—N3—C10—C11	−100.0 (3)
C4—C5—C6—C8	−179.8 (2)	C11'—C10—C11—C13	−60.3 (12)
C5—C6—C7—C2	1.7 (3)	N3—C10—C11—C13	44.0 (12)
C8—C6—C7—C2	−179.77 (19)	C11'—C10—C11—C12	72.9 (5)
C3—C2—C7—C6	−1.0 (3)	N3—C10—C11—C12	177.1 (4)
C1—C2—C7—C6	−179.47 (18)	N3—C10—C11'—C13'	−41.3 (10)
C9—N2—C8—O2	−8.7 (3)	C11—C10—C11'—C13'	51.2 (9)
C9—N2—C8—C6	171.83 (18)	N3—C10—C11'—C12'	−174.1 (4)
C7—C6—C8—O2	−173.1 (2)	C11—C10—C11'—C12'	−81.5 (5)
C5—C6—C8—O2	5.4 (3)	C9—N3—C14—C15	100.4 (2)
C7—C6—C8—N2	6.4 (3)	C10—N3—C14—C15	−71.8 (2)
C5—C6—C8—N2	−175.10 (19)	N3—C14—C15—C17	−178.37 (19)
C14—N3—C9—N2	15.8 (3)	N3—C14—C15—C16	−55.0 (3)

### Hydrogen-bond geometry (Å, º)

**Table:**

*D*—H···*A*	*D*—H	H···*A*	*D*···*A*	*D*—H···*A*
N1—H12···O2^i^	0.88	2.09	2.887 (2)	150
N2—H2···O1^ii^	0.88	1.97	2.797 (2)	155
N1—H11···S1^ii^	0.88	2.54	3.3908 (18)	163
C7—H7···O1^ii^	0.95	2.32	3.210 (2)	155

Symmetry codes: (i) −*x*+1/2, *y*+1/2, −*z*+1; (ii) −*x*, −*y*+1, *z*.
